# COVID-19 infection with asymptomatic or mild disease severity in young patients: Clinical course and association between prevalence of pneumonia and viral load

**DOI:** 10.1371/journal.pone.0250358

**Published:** 2021-04-21

**Authors:** Cherry Kim, Wooil Kim, Ji Hoon Jeon, Hyeri Seok, Sun Bean Kim, Hee Kyoung Choi, Young Kyung Yoon, Joon Young Song, Dae Won Park, Jang Wook Sohn, Won Suk Choi

**Affiliations:** 1 Department of Radiology, Korea University College of Medicine, Korea University Ansan Hospital, Ansan, Republic of Korea; 2 Department of Radiology and Medical Imaging, University of Virginia Health System, Charlottesville, VA, United States of America; 3 Division of Infectious Diseases, Department of Internal Medicine, Korea University College of Medicine, Korea University Ansan Hospital, Ansan, Republic of Korea; 4 Division of Infectious Diseases, Department of Internal Medicine, Korea University College of Medicine, Korea University Anam Hospital, Seoul, Republic of Korea; 5 Division of Infectious Diseases, Department of Internal Medicine, Korea University College of Medicine, Korea University Guro Hospital, Seoul, Republic of Korea; Universidad Nacional de la Plata, ARGENTINA

## Abstract

Few studies have focused on clinical courses or viral loads in young asymptomatic or mild patients with COVID-19 infection. We sought to better understand the clinical course and association between viral load and prevalence of pneumonia in young COVID-19 patients with asymptomatic or mild disease severity. In this retrospective study, 106 COVID-19 young patients with asymptomatic or mild disease severity were analyzed for clinical characteristics, clinical course, prevalence of radiologically proven pneumonia and viral load. The cut-off value of viral load for presence of pneumonia was also investigated. The mean age was 28.0±9.3 years. Eleven patients (10.4%) experienced viral remission within one week of diagnosis, but one (0.9%) transferred to the hospital due to aggravation of pneumonia. Patients with pneumonia had significantly higher viral load than those without, and the cut-off value of the Ct value for presence of pneumonia were 31.38. The patients with pneumonia had significantly slower recovery times than those without. Diarrhea was significantly more common in patients with pneumonia than patients without pneumonia. In conclusion, most young asymptomatic and mildly symptomatic patients showed stable clinical course. There were significant differences in viral load and recovery times between patients with and without pneumonia.

## Introduction

Even after coronavirus disease 2019 (COVID-19) was declared an international public health emergency by the World Health Organization [1], an increasing number of patients are infected with COVID-19 worldwide. However, according to the literature, 80% of COVID-19-infected patients have mild symptoms [2–5]. In the Republic of Korea, after sharp increase of COVID-19 in Daegu and the surrounding North Gyeongsang Province in March 2020, the government focused on management of asymptomatic or mild cases instead of severe patients, representing an attempt to best allocate limited medical resources in the face of the worsening public health emergency. At that time, because of the large number of new infections from patients traveling from overseas with asymptomatic or mild disease, the government’s focus was on thoroughly monitoring and quarantining people entering the country. A policy was established to quarantine all COVID-19 infected patients who traveled from abroad with asymptomatic or mild disease not requiring oxygen therapy according to certain disease severity criteria to out-of-hospital community treatment centers (CTCs). Similar policies may be required in other countries to accommodate the growing number of COVID-19 patients, regardless of whether the infections developed abroad or domestically.

Most studies have researched clinically severe patients, and few have focused on clinical course or viral load in asymptomatic or mild patients with COVID-19 infection. Previously, Liu et al. suggested a higher mean viral load in severe COVID-19 infection compared to patients with mild symptoms, suggesting an association between higher viral load and severe clinical outcomes [6]. From this, we hypothesized that there is a correlation between viral load and likelihood of developing pneumonia even among young mildly symptomatic patients, and it is important to determine when to perform an imaging exam to detect pneumonia using the criterion of viral load. Also, studying the clinical course and symptoms in young patients with mild disease will be critical in medical resource allocation.

The cycle threshold value (Ct value) is defined as the number of cycles required for the fluorescence signal to cross the threshold, and therefore it reflects viral load. Clinical samples from COVID-19 patients could be quantified using Ct value, and if Ct value is <40, samples is considered positive [7]. In the previous study, Ct values of severe COVID-19 infection was significantly lower than those of mild cases [6].

Therefore, we sought to better understand the clinical course and association between prevalence of pneumonia and viral load in young COVID-19 patients with asymptomatic or mild disease severity.

## Materials and methods

This study was approved by the Institutional Review Board of Korea University Ansan Hospital, and informed consent for use of clinical data was waived (approval number: 2020AS0213).

### Study population

This retrospective study was performed at the Korea SMEs & Startups Agency (KOSME) CTCs in Ansan, which was a designated facility to manage 115 patients with COVID-19 with asymptomatic or mild disease severity by the Korea Centers for Disease Control and Prevention (KCDC) between March 23 and May 10, 2020. Patients in this facility were all Korean, but they had returned from outside Korea and were diagnosed with COVID-19 by real-time reverse-transcriptase polymerase-chain reaction (rRT-PCR) at the airport quarantine station upon arrival in Korea. Patients were defined as having asymptomatic or mild COVID-19 according to the KCDC severity criteria (http://ncov.mohw.go.kr/en/). Asymptomatic patients were defined as a) aged <65 years, b) no underlying conditions, c) non-smoker, and d) body temperature <37.5°C without antipyretic drugs. Mildly symptomatic patients were defined as a) aged <50 years, b) >1 underlying condition, and c) a temperature <38°C with antipyretic drugs. These definitions were established to allocate “asymptomatic” and “mild” patients not requiring oxygen therapy to CTC, and those with significant symptoms and requiring oxygen therapy to specialized medical institutions. Therefore, patients with clinical signs such as tachypnea or dyspnea that might require oxygen therapy were transferred to hospitals rather than CTCs and were not included in this study.

Among these patients, those with less than two rRT-PCR results or chest radiograph (CXR) (n = 9) were excluded. As a result, 106 young COVID-19 patients with asymptomatic or mild disease severity were finally included. The mean age of the patients was 28.0±9.3 years, and 43.4% were male.

### Collection and analysis of respiratory specimens

In all patients, respiratory specimens including upper respiratory specimens in nasopharyngeal/oropharyngeal swab were collected and tested by rRT-PCR assay at the time of entering CTC. Then, rRT-PCR analysis was performed for detecting SARS-CoV-2 using AllplexTM 2019-nCoV Assay (Seegene, Seoul, South Korea). The following day, those who showed Ct values were identified. Medical laboratory experts interpreted results as negative, positive, or inconclusive. In cases of positive results, samples were collected and tested once a week. Patients with negative specimens were re-tested on the following day, while patients with inconclusive results were re-tested after two days [8]. Patients were discharged from CTC only after two consecutive negatives were confirmed at rRT-PCR intervals of 24 hours or longer.

### CXRs

CXRs were performed in all patients at the time of admission to CTC. If pneumonia was detected in CXR, the test was repeated two days later. If initial CXR was normal, the test was repeated one week later and at the time of discharge. CXR was also performed when the patient complained of dyspnea or chest pain. All CXRs were analyzed by two expert chest radiologists with 10 and 8 years of experience in consensus (C.K. and W.K.). When it was unclear whether it was pneumonia or not, the decision was made through several conferences of clinicians and radiologists based on clinical and radiological findings in consensus. Duration of pneumonia was defined as the period between the day of detection and the last day of pneumonia positivity in CXR.

In the CTCs, asymptomatic patients were observed without special treatment even if pneumonia was detected in CXRs. If a patient had symptoms such as dyspnea or tachypnea and needed oxygen therapy, they were transferred to the hospital without delay.

### Statistical analysis

Baseline characteristics of age, male sex, initial symptoms, underlying disease, continents where the patients had traveled, days between initial symptoms to diagnosis, and days from diagnosis to last follow up were summarized using descriptive statistics of proportion or mean±standard deviation (SD). To compare clinical course, viral loads, initial symptoms, and continents of travel between patients with and without radiologically proven pneumonia and between asymptomatic patients and those with mild disease, Student’s t-test was used for continuous variables and the chi-square test for categorical variables. Inter-observer agreement was assessed with weighted kappa statistics. These results were interpreted as follows: < 0.2, poor agreement; 0.21–0.4, fair agreement; 0.41–0.6, moderate agreement; 0.61–0.8, good agreement; and > 0.80, very good agreement. The diagnostic performances of the Ct value for presence of pneumonia were analyzed by receiver operating characteristic (ROC) curves and area under the curve (AUC). A *P*-value <0.05 was considered significant. All statistical analyses were performed with SPSS package, version 20.0 (SPSS, Chicago, IL)

## Results

### Clinical characteristics of patients

The clinical characteristics of patients with young asymptomatic and mildly symptomatic COVID-19 are described in **Table 1**. The mean number of days from initial symptoms to diagnosis was 12.5±11.0 days, and that from diagnosis to discharge was 24.9±11.2 days. While 16 patients (15.1%) had no symptoms, among the symptomatic patients, the most common initial symptoms were cough (35.8%) and fever (34%). Patients without underlying disease represented 75.5% of the total. The most common continent of travel was America (54.7%), followed by Europe (32.1%) and Asia (13.2%).

**Table 1:** Baseline characteristics, initial symptoms, underlying disease, and continents of travel in young COVID-19 patients with asymptomatic or mild disease severity.

**Baseline characteristics**	
Age, years (median, interquartile range)	28.0±9.3 (26, 22.8–30.0)
Male sex, n (%)	46 (43.4)
Days from initial symptoms to diagnosis (median, interquartile range)	12.5±11.0 (10, 9.0–15.0)
Days from diagnosis to discharge (median, interquartile range)	24.9±11.2 (25, 16.0–34.0)
**Initial symptoms, n (%)**	
Asymptomatic	16 (15.1)
Cough	38 (35.8)
Fever	36 (34.0)
Sore throat	26 (24.5)
Headache	20 (18.9)
Hyposmia	19 (17.9)
Rhinorrhea	18 (17.0)
Sputum	8 (7.5)
Muscle pain	7 (6.6)
Diarrhea	7 (6.6)
Chest pain	2 (1.9)
Ocular pain	1 (0.9)
**Underlying disease, n (%)**	
None	80 (75.5)
Rhinitis	9 (8.5)
Asthma	7 (6.6)
Migraine	2 (1.9)
Iron deficiency anemia	2 (1.9)
Atopic dermatitis	2 (1.9)
Hyperlipidemia	1 (0.9)
Endometriosis	1 (0.9)
Depression disorder	1 (0.9)
Hair loss	1 (0.9)
**Continent of travel, n (%)**	
America	58 (54.7)
Europe	34 (32.1)
Asia	14 (13.2)

Note—COVID-19, coronavirus disease 2019; rRT-PCR, real-time reverse-transcriptase polymerase-chain reaction; Ct value, cyclic threshold value.

### Clinical course, viral load, and prevalence of radiologically proven pneumonia

**Table 2** summarizes clinical course, viral load, and prevalence of radiologically proven pneumonia in young COVID-19 patients with asymptomatic or mild disease severity. The two radiologists were in very good agreement for the presence of pneumonia (kappa = 0.962).

**Table 2:** Clinical course, viral load, and prevalence of radiologically proven pneumonia in young COVID-19 patients with asymptomatic or mild disease severity.

	N = 106
**Clinical course**	
Two consecutive rRT-PCR negative results within one week, n (%)	11 (10.4)
Days from diagnosis to two consecutive negative rRT-PCR (median, interquartile range)	20.0±9.2 (19, 27.5–33.0)
**Viral load**	
The Ct value at the time of entering CTC (median, interquartile range)	33.6±5.5 (33.3, 28.8–33.8)
**Development of pneumonia**	
Pneumonia detected in chest radiograph, n (%)	48 (45.3)
Days from diagnosis to pneumonia detection (median, interquartile range)	3.4±5.5 (2.0, 1.0–4.0)
Pneumonia duration, days (median, interquartile range)	12.9±8.0 (10, 6.3–18.0)
The lowest Ct value during pneumonia (median, interquartile range)	30.2±4.2 (31.1, 27.5–33.0)

Note—COVID-19, coronavirus disease 2019; rRT-PCR, real-time reverse-transcriptase polymerase-chain reaction; CTC, community treatment center; Ct value, cyclic threshold value.

Among 106 patients, one (0.9%) transferred to the hospital due to aggravation of COVID-19 pneumonia and symptoms. All patients including the one transferred to the hospital achieved virologic remission and were discharged from CTCs. No patient died from COVID-19 infection. Eleven patients (10.4%) showed two consecutive rRT-PCR negative results indicating viral remission within one week from diagnosis. The mean number of days from diagnosis to two consecutive rRT-PCR negative test was 20.0±9.2 in all patients. The mean Ct values at the time of entering CTC was 33.6±5.5.

Among 106 patients, pneumonia was detected during disease course on CXRs of 48 patients (45.3%). The mean number of days from diagnosis to pneumonia detection was 3.4±5.5 days. The mean duration of pneumonia was 12.9±8.0 days. The mean lowest Ct value during pneumonia was 30.2±4.2.

### Comparisons between patients with and without pneumonia

Comparisons of clinical course, viral loads, symptoms, and continents of travel between young COVID-19 patients with and without radiologically proven pneumonia and asymptomatic or mild disease severity are shown in **Table 3**. There were no significant differences of age, male sex, and continents of travel between the two groups (all *P*>0.05).

**Table 3:** Comparisons of clinical course, viral load, symptoms, and continents of travel between young COVID-19 patients with and without radiologically proven pneumonia and asymptomatic or mild disease severity.

	Without pneumonia (n = 58)	With pneumonia (n = 48)	P-value
Age, years (median, interquartile range)	26.7±7.9 (25.0, 22.8–29.3)	29.5±10.7 (28.0, 22.3–31.5)	0.130
Male sex, n (%)	25 (43.1)	21 (43.8)	0.540
The Ct value obtained at the time of entering CTC (median, interquartile range)	35.1±5.0 (35.3, 30.4–36.5)	31.9±5.5 (33.3, 27.4–32.8)	0.001
Days from initial symptoms to diagnosis (median, interquartile range)	15.1±11.7 (13.0, 7–19.5)	9.3±9.2 (8.0, 3.0–13.0)	0.008
Days from diagnosis to two consecutive negative rRT-PCR (median, interquartile range)	18.6±9.7 (17.0, 8.5–26.8)	22.8±7.1 (23.5, 17.8–26.3)	0.040
Two consecutive negative rRT-PCR within a week (%) (median, interquartile range)	11 (19.0)	0	0.001
Two consecutive negative rRT-PCR within two weeks (%) (median, interquartile range)	16 (27.6)	5 (10.4)	0.030
**Initial symptoms, n (%)**			
None	10 (17.2)	6 (12.5)	0.592
Cough	21 (36.2)	17 (35.4)	0.078
Sputum	4 (6.9)	4 (8.3)	>0.999
Chest pain	1 (1.7)	1 (2.1)	>0.999
Headache	11 (19.0)	9 (18.8)	>0.999
Fever	19 (32.8)	17 (35.4)	0.838
Sore throat	13 (22.4)	13 (27.1)	0.653
Hyposmia	14 (24.1)	5 (10.4)	0.079
Diarrhea	1 (1.7)	6 (12.5)	0.045
Ocular pain	1 (1.7)	0	>0.999
Rhinorrhea	9 (15.5)	9 (18.8)	0.796
Muscle pain	2 (3.4)	5 (10.4)	0.240
**Continent of travel**			
America	28 (48.3)	30 (62.5)	0.337
Europe	21 (36.2)	13 (22.4)	
Asia	9 (15.5)	5 (10.4)	

Note—COVID-19, coronavirus disease 2019; CTC, community treatment center; rRT-PCR, real-time reverse-transcriptase polymerase-chain reaction; Ct value, cyclic threshold value.

The Ct value at the time of entering CTC of patients with pneumonia was significantly lower than that of patients without pneumonia (31.9±5.5 vs. 35.1±5.0, *P* = 0.001). The mean number of days from initial symptoms to diagnosis of patients with pneumonia was significantly smaller than that of patients without pneumonia (9.3±9.2 days vs. 15.1±11.7 days, *P* = 0.008). The mean number of days from diagnosis to two consecutive negative rRT-PCR was significant smaller in patients without pneumonia than in patients with pneumonia (18.6±9.7 days vs. 22.8±7.1 days, *P* = 0.040). Two consecutive rRT-PCR negative were confirmed within a week in 11 patients without pneumonia, whereas none of patients with pneumonia did not obtain two consecutive rRT-PCR negative within a week (*P* = 0.001). In addition, there were significantly more patients who were confirmed at two consecutive rRT-PCR negative within two weeks in patients without pneumonia than in patients with pneumonia (16 [27.6%] vs. 5 [10.4%], *P* = 0.030).

Among initial symptoms, diarrhea was significantly more common in patients with pneumonia than patients without pneumonia (6 [12.5%] vs. 1 [1.7%], *P* = 0.045). No other symptoms showed significant difference between the groups (all *P*>0.05).

### Cut-off values of Ct values for developing pneumonia

**Fig 1:**
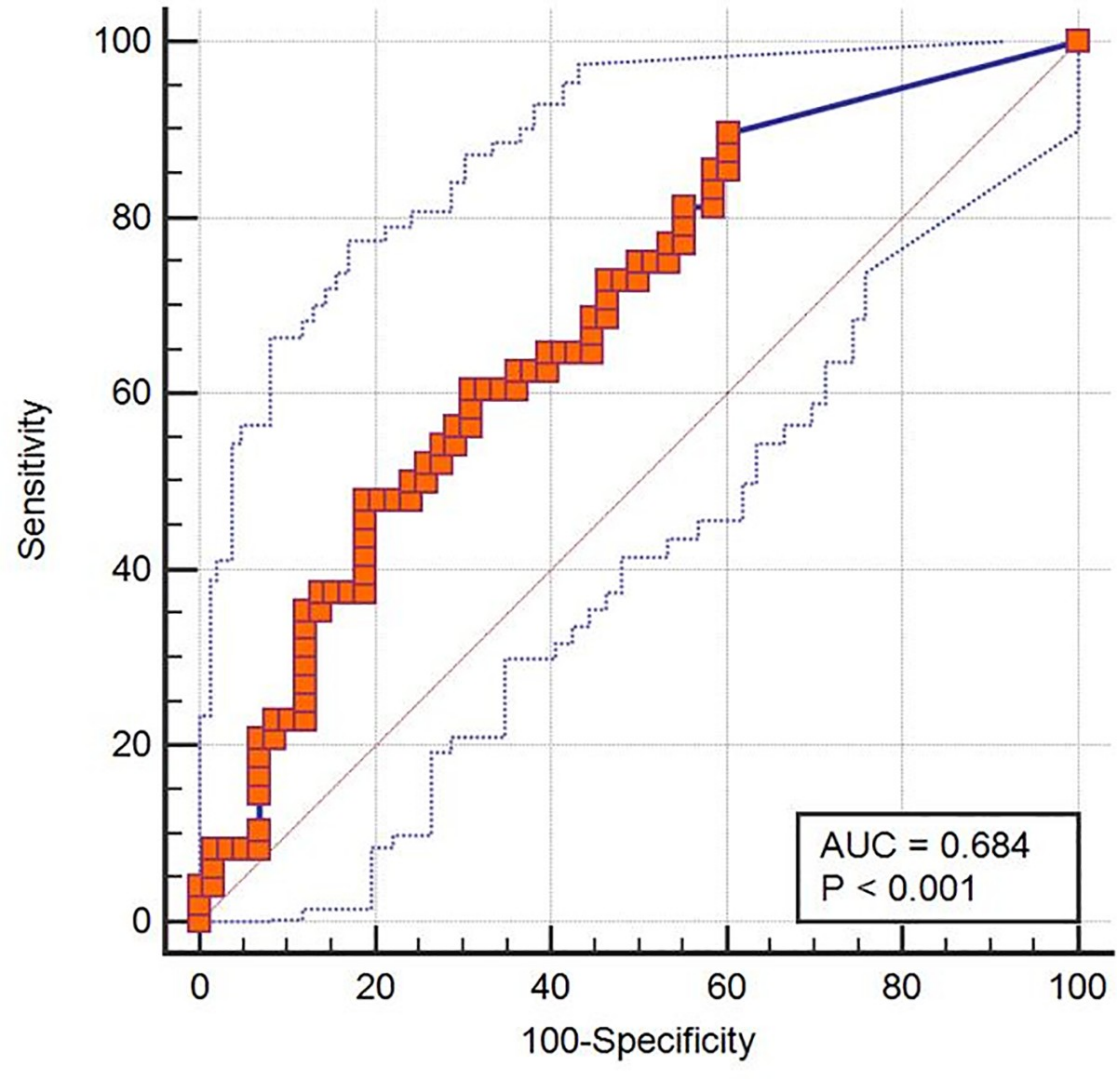
The ROC curve of the Ct value obtained at the time of entering community treatment center for presence of pneumonia. The AUC of the ROC curve of the Ct value obtained at the time of entering community treatment center for presence of pneumonia was 0.684 (95% confidence interval [CI], 0.587 to 0.771; *P*<0.001).

The AUC of the ROC curve of the Ct value at the time of entering CTC for presence of pneumonia was 0.684 (95% confidence interval [CI], 0.587 to 0.771; P<0.001) (**Fig 1**). The cut-off value of the Ct value at the time of entering CTC for presence of pneumonia was 31.38 (sensitivity and specificity, 62.5% and 65.5%, respectively).

### Comparisons between asymptomatic patients and those with mild disease

**Fig 2:**
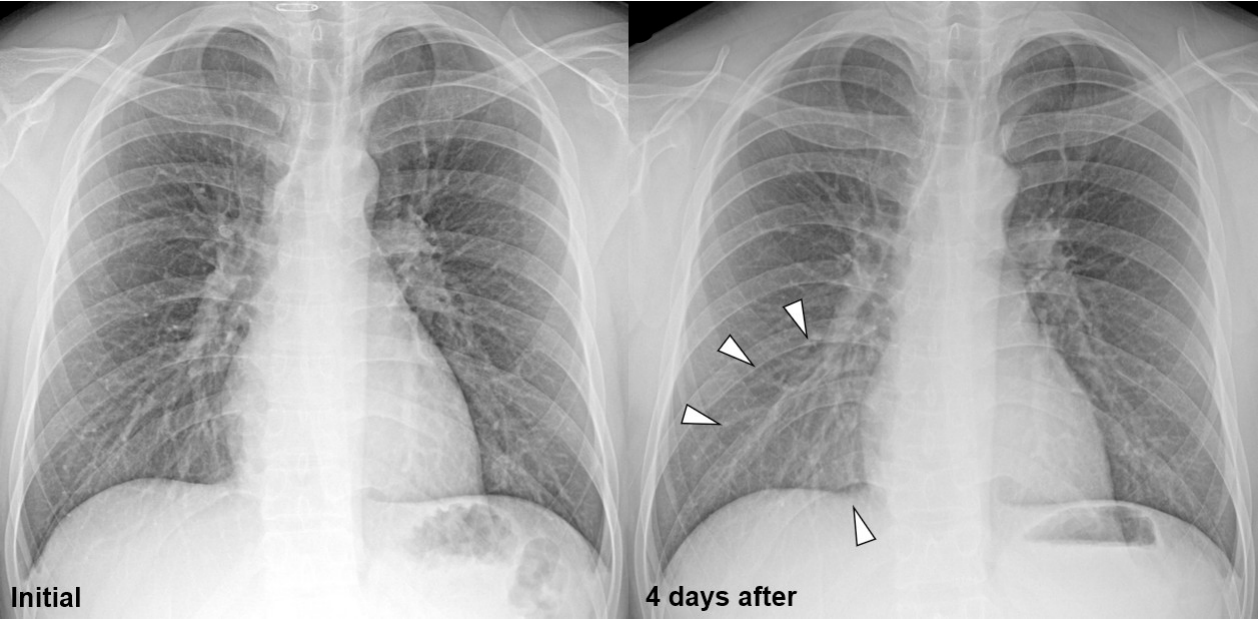
Chest radiographs (CXRs) of an initially asymptomatic patient. The CXR of a 22-year-old asymptomatic male patient. This patient had chest pain after four days, and CXR was performed. Compared with the initial CXR, increased opacity was detected in the right lower lung zone (arrowheads), suggestive of pneumonia. However, the patient’s symptoms were not aggravated and the medical staff decided to observe this patient in CTC, rather than transfer him to a hospital. The opacity had disappeared at the time of discharge.

**Fig 3:**
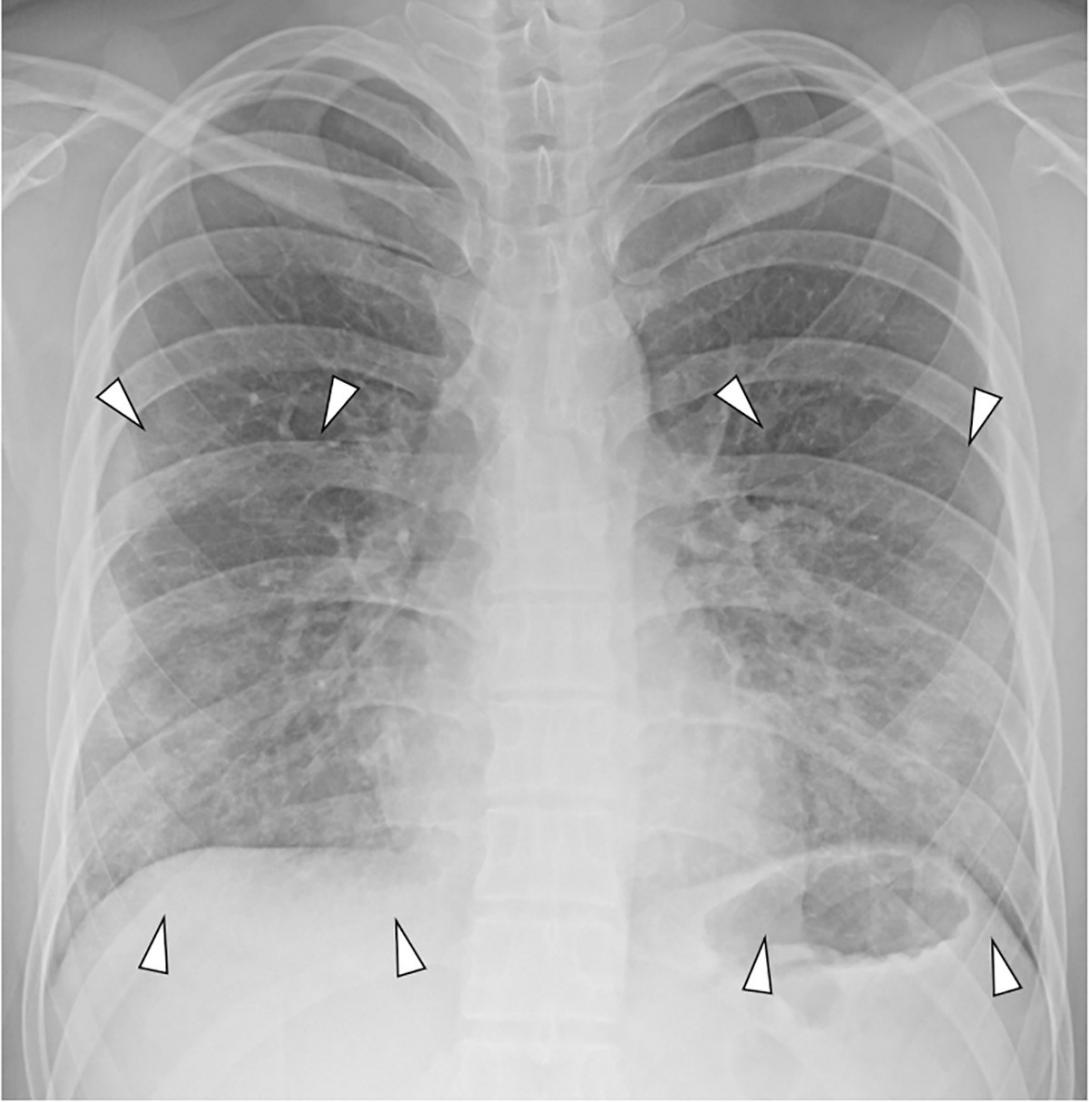
Chest radiograph (CXR) of a patient with mild COVID-19 disease. The initial CXR of a 29-year-old male patient with mild symptom. The patient reported rhinorrhea and chest pain at the time of CTC entry. However, the initial CXR showed diffusely increased opacities in both the mid- to lower-lung zones (arrowheads). Dyspnea was aggravated the following day, and the patient was transferred to a hospital.

Comparisons of the clinical course, viral loads, symptoms, and continents of travel between asymptomatic patients and those with mild COVID-19 disease are shown in **Table 4**. There were no significant differences in the age, sex, and continent of travel between the two groups (all *P*>0.05). Pneumonia was detected on CXR in six asymptomatic patients (6/16, 37.5%) and 42 patients with mild disease (42/90, 46.7%); however, there was no statistical significance (*P* = 0.591) (**Figs 2** and **3**). The Ct value of mild patients at the time of CTC entry was lower than that of asymptomatic patients without statistical significance (33.5±5.6 vs. 34.7±4.7, *P* = 410).

**Table 4:** Comparisons of the clinical course, viral load, symptoms, and continents of travel between asymptomatic COVID-19 patients and patients with mild disease severity.

	Asymptomatic patients (n = 16)	Mild patients (n = 90)	P-value
Age, years (median, interquartile range)	29.3±11.9	27.7±8.8	0.528
	(27.0, 22–33)	(26.0, 23–30)	
Male sex, n (%)	10 (62.5)	36 (40.0)	0.094
The Ct value obtained at the time of entering CTC (median, interquartile range)	34.7±4.7	33.5±5.6	0.410
	(35.4, 30.8–39.8)	(33.2, 30.3–39.8)	
Days from initial symptoms to diagnosis (median, interquartile range)	13.2±12.2	12.4±10.8	0.803
	(12.0, 30.8–40.0)	(10. 0–54.0)	
Days from diagnosis to two consecutive negative rRT-PCR (median, interquartile range)	18.7±7.5	20.6±9.2 (19.0,	0.529
	(21.0, 14.0–25.0)	14.0–27.0)	
Two consecutive negative rRT-PCR within a week (%)	2 (12.5)	9 (10.0)	0.763
Two consecutive negative rRT-PCR within two weeks (%)	4 (19.8)	17 (18.9)	0.517
**Continent of travel**			
America	11 (68.8)	47 (52.2)	0.301
Europe	3 (18.8)	35 (38.9)	
Asia	2 (12.5)	8 (8.9)	

Note—COVID-19, coronavirus disease 2019; CTC, community treatment center; rRT-PCR, real-time reverse-transcriptase polymerase-chain reaction; Ct value, cyclic threshold value.

## Discussion

The present study showed the clinical course including viral load and association between viral load and prevalence of pneumonia of young COVID-19 patients with asymptomatic or mild disease severity managed in an out-of-hospital CTC. Eleven patients (10.4%) achieved viral remission within one week, but one patient (0.9%) transferred to the hospital due to aggravation of pneumonia and symptoms. Pneumonia was detected in 45.3% of patients during follow up and all achieved virologic remission and were discharged after viral clearance. However, significantly higher viral load was shown in patients with pneumonia than in patients without pneumonia, and days to virologic remission were significantly shorter in patients without pneumonia than in patients with pneumonia. Diarrhea was significantly more common in patients with pneumonia than in patients without pneumonia, and the cut-off value of the Ct value at the time of entering CTC for presence of pneumonia was 31.38. There were no significant differences in pneumonia development between asymptomatic and mild patients.

In this study, most asymptomatic and mildly symptomatic patients showed relatively stable clinical course, in accordance with previous studies [9]. This indicates that out-of-hospital CTC and their strategies can manage asymptomatic and mild patients well and can properly identify patients with severe symptoms by monitoring time of exacerbation from mild to severe.

Semiquantitative testing using rRT-PCR with respiratory specimens has been widely used to diagnose COVID-19, and nasopharyngeal/oropharyngeal swabs are usually obtained to monitor serial viral load. However, the viral load collected from the upper respiratory tract does not directly reflect infection of the lower respiratory tract (i.e., viral pneumonia). As a method to identify viral pneumonia, radiologic imaging studies such as CXRs or computed tomography (CT) should be performed. Since medical resources are limited, it is important to determine when imaging studies should be conducted in symptomatic or mild patients. Therefore, we suggest a cut-off value of Ct value for presence of pneumonia for efficient allocation of medical resources. Since a low Ct value further suggests the possibility of viable virus and presence of pneumonia, we recommend that patients with a lower Ct value than a cut-off value have a difference in the isolation period even if they do not have symptoms.

In our results, the Ct value at the time of entering CTC was significantly lower than those of patients without pneumonia. The mean numbers of days to two consecutive rRT-PCR negative of patients with pneumonia were significantly longer than those in patients without pneumonia. In addition, none of patients with pneumonia obtained with two consecutive rRT-PCR negative within a week, whereas there were 19% of patients without pneumonia who confirmed at two consecutive rRT-PCR negative within a week. Only 10.4% of patients with pneumonia obtained with two consecutive rRT-PCR negative within two weeks. In addition to previous study revealing that viral load was significantly higher in severely symptomatic patients than in mildly symptomatic patients [6], our results showed significant difference of viral load between patients with and without lower respiratory tract infection among young asymptomatic or mildly symptomatic patients, and revealed that young patients with pneumonia had significantly slower recovery times than those without. In addition, the Ct value at the time of entering CTC of mild patients was also lower than that of asymptomatic patients, although this was not statistically significant.

Diarrhea was the only initial symptom significantly more common in patients with pneumonia than in patients without pneumonia. In our study, among seven patients with diarrhea as the initial symptom, six (85.7%) also had respiratory symptom. However, in this patient who had only diarrhea, COVID-19 pneumonia developed at 21 days from the initial symptom. Previously, Han et al. reported that patients with viral load detected in stool are more likely to have a longer delay before viral remission compared to patients with respiratory symptoms but no digestive symptoms, suggesting higher viral burden in these patients [10]. Also, a prospective study by Park et al. suggested that the Ct values of respiratory specimens may predict positive findings in feces, because patients with positive fecal samples showed lower Ct values in the upper respiratory tract than did patients with negative fecal samples [11]. These results suggest that diarrhea might reflect higher viral burden of COVID-19 infection and could be associated with lower respiratory infection of COVID-19. However, despite the statistical significance of our results, our study’s clinical power is in question due to the relatively small number of patients. Further research is warranted.

There are several limitations to this study. First, it was retrospective in nature, and nine patients who did not have enough study information were excluded. In addition, patients did not have a uniform period from symptoms to diagnosis. Because all patients returned from abroad, it was difficult to obtain an early confirmatory examination depending on the country of travel; in some cases, COVID-19 was not suspected if the symptoms were mild. Therefore, it was difficult to estimate the exact onset time and instead considered onset as diagnosis at the airport. Also, the viral load in rRT-PCR tested at the airport was unknown, and there was limited data on the duration of symptoms and symptoms at the time of pneumonia diagnosis. It is also possible that mild pneumonia not detectable in CXR might have been missed. However, all CXR results were read by two expert chest radiologists and were retrospectively reanalyzed for accuracy.

In conclusion, most young asymptomatic and mildly symptomatic patients managed in an out-of-hospital CTC showed stable clinical course. There was significant difference in viral load and recovery time was significant difference between patients with and without pneumonia. A cut-off value of Ct value for presence of pneumonia was also suggested. In addition, patients with pneumonia have more frequent diarrhea than patients without.

## Supporting information

S1 File Minimal data of our results. (XLSX)
